# Steroid biotransformations in biphasic systems with *Yarrowia lipolytica* expressing human liver cytochrome P450 genes

**DOI:** 10.1186/1475-2859-11-106

**Published:** 2012-08-09

**Authors:** Andreas Braun, Martina Geier, Bruno Bühler, Andreas Schmid, Stephan Mauersberger, Anton Glieder

**Affiliations:** 1 Institute of Molecular Biotechnology, Graz University of Technology, ACIB GmbH, Petersgasse 14, Graz, Austria; 2 Laboratory of Chemical Biotechnology, TU Dortmund University, Emil-Figge-Strasse 66, Dortmund 44227, Germany; 3 Institute of Microbiology, Dresden University of Technology, Dresden 01062, Germany

**Keywords:** *Yarrowia lipolytica*, Biphasic sytem, Cytochrome P450, Steroid, Whole-cell bioconversion

## Abstract

**Background:**

*Yarrowia lipolytica* efficiently metabolizes and assimilates hydrophobic compounds such as n-alkanes and fatty acids. Efficient substrate uptake is enabled by naturally secreted emulsifiers and a modified cell surface hydrophobicity and protrusions formed by this yeast. We were examining the potential of recombinant *Y. lipolytica* as a biocatalyst for the oxidation of hardly soluble hydrophobic steroids. Furthermore, two-liquid biphasic culture systems were evaluated to increase substrate availability. While cells, together with water soluble nutrients, are maintained in the aqueous phase, substrates and most of the products are contained in a second water-immiscible organic solvent phase.

**Results:**

For the first time we have co-expressed the human cytochromes P450 2D6 and 3A4 genes in *Y. lipolytica* together with human cytochrome P450 reductase (hCPR) or *Y. lipolytica* cytochrome P450 reductase (YlCPR). These whole-cell biocatalysts were used for the conversion of poorly soluble steroids in biphasic systems.

Employing a biphasic system with the organic solvent and *Y. lipolytica* carbon source ethyl oleate for the whole-cell bioconversion of progesterone, the initial specific hydroxylation rate in a 1.5 L stirred tank bioreactor was further increased 2-fold. Furthermore, the product formation was significantly prolonged as compared to the aqueous system.

Co-expression of the human CPR gene led to a 4-10-fold higher specific activity, compared to the co-overexpression of the native *Y. lipolytica* CPR gene. Multicopy transformants showed a 50-70-fold increase of activity as compared to single copy strains.

**Conclusions:**

Alkane-assimilating yeast *Y. lipolytica*, coupled with the described expression strategies, demonstrated its high potential for biotransformations of hydrophobic substrates in two-liquid biphasic systems. Especially organic solvents which can be efficiently taken up and/or metabolized by the cell might enable more efficient bioconversion as compared to aqueous systems and even enable simple, continuous or at least high yield long time processes.

## Introduction

Cytochrome P450s (CYPs) are a large, ubiquitous family of heme-containing monooxygenases that are responsible for the oxidative metabolism of a wide variety of drugs, environmental chemicals and endogenous compounds, such as steroids, prostaglandins and fatty acids [1].

Most cytochrome P450 systems are composed of a monooxygenase and one or two additional proteins, constituting an electron transfer chain. Genes encoding these components are either expressed individually or linked resulting in self sufficient CYPs. To some extent, the natural electron transport chain from NAD(P)H to the heme containing cytochrome P450 can be replaced by either homologues or different proteins with similar function e.g. flavodoxin and flavodoxin reductase to support catalytic activity [2]. Therefore, the activity of CYPs is not only determined by its abundance, but also by the abundance of the electron transport partners [3] and possibly by their molar ratio.

Eukaryotic CYPs are membrane associated and many of those are located on the cytosolic side of the endoplasmic reticulum membrane. However, several important CYPs such as the vitamin D3 25-hydroxylase CYP27B1 are also associated to mitochondrial membrane [4]. In mammalian cells, expression takes place in different tissues, but the highest levels are found in the liver, where CYPs have the principal function to introduce an oxygen atom into hydrophobic substrates. The increased hydrophilicity of the product facilitates its elimination from the mammalian body.

Several genes of xenobiotic-metabolizing CYPs are expressed in human liver, among which CYP1A2, CYP2C9, CYP2C19, CYP2D6 and CYP3A4 appear to be most commonly responsible for drug metabolism [5]. The relative importance for drug metabolism is reflected in the abundance of these enzymes, e.g., CYP3A4 being most abundant with ~ 30% of total CYP in liver cells, and the preference to bind and/or metabolize chemicals with structures commonly found in drugs, e.g., CYP2D6 preferentially binds widely used drugs with basic amine functions [3].

Many of these drug-metabolizing CYPs are subject to polymorphisms, duplications, and differential expression levels, which gives rise to wide variation in pharmacokinetics profiles. This makes CYPs highly important to the pharmaceutical industry, where human drug metabolites are essential for drug development and analysis.

In order to provide sufficient amounts of human CYPs, several recombinant expression systems have been investigated in the past 20 years, from the more complicated and expensive mammalian [6] and *Baculovirus*-infected insect cell system [7] to the “simpler” expression host *Escherichia coli*. Since the first functional expression of mammalian CYPs in bakers’s yeast Saccharomyces cerevisiae was demonstrated in the mid 80s [8,9], in recent years, the focus shifted more and more to yeast systems, which combine the ease of handling of prokaryotic systems with the sub-cellular structure of eukaryotic systems reassembling a more natural environment. Mammalian CYP genes have been expressed, e.g., in the yeasts *S. cerevisiae*[8-14], *P. pastoris*[15,16], *S. pombe*[17-19], and *Y. lipolytica*[20-22]. Frequently, whole-cells were used for drug metabolite synthesis to deal with inherent stability problems of human CYP enzymes and regeneration of NADPH.

Most of the typical substrates for CYPs are very hydrophobic and most probably enter the enzyme’s active site via biological membranes. This also explains why specific activities of truncated and soluble human CYPs are lower than those of their native membrane bound counterparts [23]. Another obstacle in performing bioconversions with hydrophobic substrates is their very low solubility in the aqueous phase, which limits cellular uptake and thus overall biotransformation performance. Investigations showed that the substrates have to be added in concentrations above the solubility limit to achieve efficient biotransformation. To ensure a homogenous suspension, the substrates have to be dissolved at high concentration in an organic solvent and quickly added to the aqueous solution [24].

However, there are several approaches to increase substrate availability. The addition of water-miscible organic solvents or detergents has been tested [17], which may results in a drastic increase of substrate solubility. Some solvents and most of the detergents, however, have a strong impact on cell membranes, compromising cell integrity and viability, and thus biocatalyst functionality.

In a more sophisticated approach, water-soluble cyclodextrins, toroid-shaped cyclic oligosaccharids, have been used to capture hydrophobic substances in their cavities, thereby increasing their apparent solubility. This approach has been successfully used with *Mycobacterium sp.*[25], but seems to have toxic effects on yeast cells [17].

Biphasic systems consisting of an organic and an aqueous phase, represent a valuable tool for the biotransformation of hydrophobic substrates [26-32]. The organic phase regulates the substrate and product concentration in the aqueous phase, allowing high overall concentrations of otherwise toxic hydrophobic substrates. Furthermore, such a system can be used to avoid inhibition effects by substrate and/or product and can help to guide equilibrium reactions into the desired direction enhancing stereoselectivity. A crucial step is the choice of the organic phase depending on different parameters, including toxic or inhibitory effects of the solvent on the cells as well as substrate and product solubility [31,32].

Our studies have been focused on *Y. lipolytica* which is naturally adapted to such two-phase systems. *Y. lipolytica* is often isolated from biphasic environments like dairy products rich in lipids [33] or oil-polluted soil and water [34,35]. Applications of *Y. lipolytica* include bioremediation of diesel-contaminated soils [36] and olive-mill waste water [37], protein production on alkanes [38], and aroma compound formation from fatty acid derivatives [39]. When *Y. lipolytica* is grown on hydrophobic substrates, the cell surface is in direct contact with substrate droplets and several modifications in cell structure occur, which are probably related to hydrophobic substrate transport. These observations have led to the hypothesis that hydrophobic substrates can migrate through channels via the plasma membrane to the ER [40,41].

These unique properties of the alkane utilizing yeast *Y. lipolytica* together with the availability of efficient genetic tools for this species underscore its potential for biotransformations in biphasic systems. The yeast *Y. lipolytica* has been revealed as one of the most suitable host for heterologous protein production [42] and several CYP genes of mammalian e.g. from *Bos taurus* and *Homo sapiens s*. [20-22], plant e.g. green bell pepper [43], and fungal origin e.g. *Candida maltosa* and *Rhodotorula minuta*[44,45], have been expressed in this yeast so far.

In this study, we report the first example of functional co-expression of genes encoding the important human liver CYPs CYP2D6 or CYP3A4 together with hCPR and intrinsic YlCPR in *Y. lipolytica* and their use in biphasic whole-cell bioconversions aiming at steroid oxidation and as a model for the hydroxylation of other poorly soluble substrates.

## Results and discussion

### Cloning and expression of genes for human liver cytochrome P450s and CPRs

One of the most frequently studied bottlenecks in heterologous gene expression is the variable codon-usage and -bias of different organisms. By exchanging rare codons with more frequently used ones of the host, the expression of heterologous genes can be significantly improved in some cases [46]. In some cases, however native genes provided better results than codon optimized variants. In this study, we compared expression of wild-type and codon optimized genes for CYP2D6, CYP3A4 and human CPR in *Y. lipolytica*.

Using *Y. lipolytica*, it is possible to create multicopy integration clones employing the auxotrophic marker *ura3* linked to a largely truncated and deficient promoter [47,48]. This approach previously allowed the human CYP1A1, YlCPR and bovine CYP17 gene expression to be increased several fold [22,49,50].

**Figure 1:**
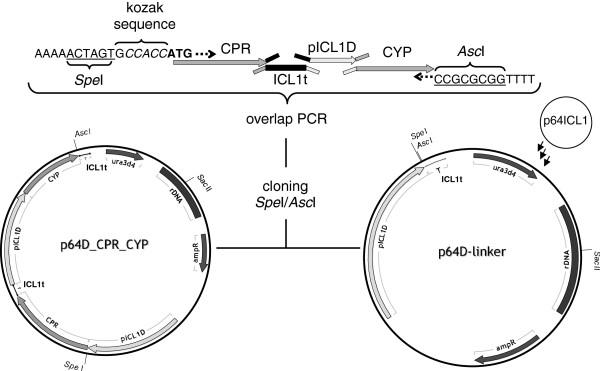
**Schematic representation of construction of the co-expression vector.** Construction of multicopy coexpression vectors by inserting overlap extension PCR fragments into the multicopy integrative vector p64D-linker containing the multicopy selective *ura3d4* marker.

In a similar approach, the genes encoding CYP2D6, CYP3A4, human cytochrome P450 reductase (hCPR) and *Y. lipolytica* cytochrome P450 reductase (YlCPR) were cloned and ligated into (i) the integrative plasmid vector p64D-linker for selection of multicopy transformants, as well as into (ii) p65D-linker promoting single copy integration. The resulting series of expression vectors contained each of the two expression cassettes under the control of the ICL1 promoter (pICL1D) and terminator (ICL1t), one for CYP and one for CPR. *Y. lipolytica* H222-S4 was transformed with the resulting *Sac*II (Figure 1) linearized plasmids.

Copy numbers of selected clones were verified by RT-PCR. Supposed single copy transformants indeed showed one integrated copy, while multicopy transformants showed between 10 and more than 40 integrated copies (Table 1) as determined by quantitative PCR, which is in good accordance with previous results [20,22,47,48,51]. 

**Table 1:** Used Strains and their expression plasmid copy number determined by RT-PCR

**Strains**	**Description**	**Copy number**	**Reference**
H222-S4	*MATA ura3-302*^a^		[76]
	(Ura^-^, Alk^+^, Tgl^+^, Lip^+^, Eth^+^, Glu^+^,Suc^+^)		
YL23	H222-S4 transformed with SacII – linearized p65D-linker plasmid, negative control		This work
YL21	H222-S4 transformed with SacII - linearized p64D-hCPRwt-2D6syn plasmid	46 ± 4	This work
YL10	H222-S4 transformed with SacII - linearized p64D-hCPRsyn-2D6syn plasmid	30 ± 3	This work
YL22	H222-S4 transformed with SacII - linearized p64D-hCPRwt-3A4syn plasmid	23 ± 2	This work
YL11	H222-S4 transformed with SacII - linearized p64D-YlCPR-2D6syn plasmid	26 ± 2	This work
YL18	H222-S4 transformed with SacII - linearized p64D-YlCPR-3A4syn plasmid	10 ± 1	This work
YL15	H222-S4 transformed with SacII - linearized p65D-hCPRwt-2D6syn plasmid	0.9 ± 0.1	This work
YL20	H222-S4 transformed with SacII - linearized p65D-hCPRwt-3A4syn plasmid	1.1 ± 0.1	This work
YL12	H222-S4 transformed with SacII - linearized p65D-YlCPR-2D6syn plasmid	1.1 ± 0.1	This work
YL19	H222-S4 transformed with SacII - linearized p65D-YlCPR-3A4syn plasmid	1.1 ± 0.1	This work

^a^*ura3-302*: *URA3* disrupted by a construct pXPR2 SUC2 (invertase gene from *S. cerevisiae*). This allele confers the ability to grow on sucrose or mollasses [76,77].

**Figure 2:**
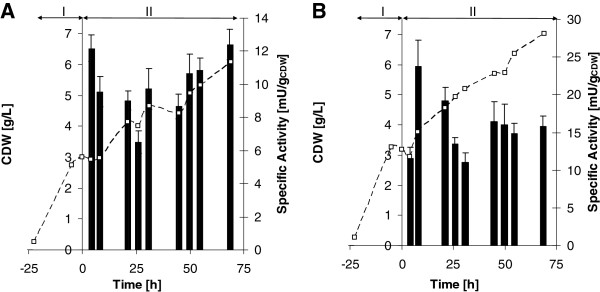
**CYP activity and *Y. lipolytica*biomass production during induction.** CYP2D6 (1’-hydroxybufuralol formation, **A**) and CYP3A4 (6β-hydroxyprogesterone formation, **B**) activity during ethanol induction of *Y. lipolytica* strains YL21 and YL22, respectively. The cultures were incubated at 28°C and induced by ethanol after glucose depletion. The Roman numerals indicate the following process phases: I) batch growth with glucose; II) induction phase of about 70 h. Squares highlight biomass concentrations determined as cell dry weight, while bars document CYP expression levels measured as specific whole-cell activity. Experiments were performed in triplicates. Samples were taken after 4,8,21,26,31,45,50,55 and 69 h.

It is common knowledge that separating biomass production and the expression phase of microbial cell cultures can be beneficial for heterologous protein production, both in terms of a reduced metabolic burden and limitation of a potentially harmful toxic activity. Therefore, *Y. lipolytica* was first grown in YNB medium with glucose as a sole carbon source (YNBG). Upon depletion of glucose and a short starvation phase, heterologous protein production in shake flasks was initiated by induction of the *ICL1* promoter with addition of ethanol. After a short lag phase, while switching from glucose to ethanol, a further increase in biomass was observed (Figure 2). Bufuralol and progesterone were used to assess CYP2D6 and CYP3A4 activity, respectively. Already after 4 to 8 h of induction, *Y. lipolytica* cells harboring CYP genes showed activity towards bufuralol or progesterone, the specific activity did not further increase and remained constant for 70 h (Figure 2). For practical reasons all further experiments were carried out with cells harvested after 26 h of induction.

**Figure 3:**
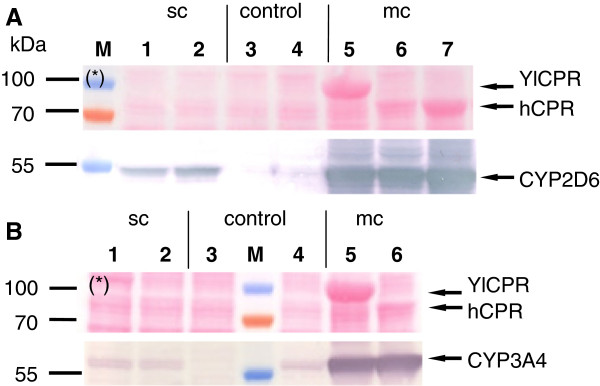
**Western blot analysis.** Western blot analysis of microsomes isolated after 26 h of ethanol induction from cultures of *Y. lipolytica* H222-S4 transformed with p64D- or p65D-based integrative vectors, containing different combinations of CYPs and CPRs. Protein transfer was monitored by Ponceau S (*) staining, where putative CPR-protein bands in multicopy transformants were already visible. CYP-protein bands CYP2D6 (**A**) and CYP3A4 (**B**) were immunodetected with anti CYP2D6 and CYP3A4 antibodies, respectively.– CYP2D6 was detected in single copy clones (sc) YL12 and YL15 expressing YlCPR-WT + 2D6syn (1), hCPR-WT + 2D6syn (2) respectively and multicopy clones (mc) YL11, YL10 and YL21 expressing YlCPR-WT + 2D6syn (5), hCPRsyn + 2D6syn (6), hCPR-WT + 2D6syn (7) respectively. CYP3A4 was detected in single copy clones (sc) YL19 and YL20 expressing YlCPR-WT + 3A4syn (1), hCPR-WT + 3A4syn (2) respectively and multicopy clones (mc) YL18 and YL22 expressing YlCPR-WT + 3A4syn (5), hCPR-WT + 3A4syn (6) respectively. YL23 clone harboring empty p65D-linker vector (3 + 4 in both panels) was used as negative control. PageRuler Prestained Protein Ladder (M) was used for monitoring protein separation and transfer efficiency.

To confirm expression of heterologous cytochrome P450s, microsomal protein of each clone sampled after 26 h of ethanol induction were analyzed by Western blotting. Bands of CYP2D6 (~ 55 kDa) and CYP3A4 (~ 57 kDa) were detected at the correct sizes. No Western blots were performed for CPR, since strong protein bands with the correct sizes for hCPR (~ 75 kDa) and YlCPR (~ 85 kDa) were already detectable by Ponceau-staining of the blots for multicopy transformants, indicating the high expression levels of CPR. (Figure 3).

Human liver cytochromes P450 2D6 and 3A4 and human cytochrome P450 reductase (hCPR) as well as *Y. lipolytica* cytochrome P450 reductase (YlCPR) were functionally expressed. The codon optimized versions of either CYPs showed best activities in combination with wild-type human CPR and wild-type *Y. lipolytica* CPR (data not shown). Therefore, these combinations were used for Western blot analysis and further used throughout this study.

**Figure 4:**
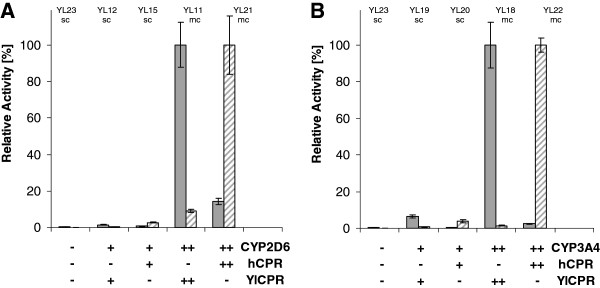
**Influence of copy number and type of reductase on CYP activity.** CYP2D6 (**A**) and CYP3A4 (**B**) activity of and CPR activities of *Y. lipolytica* single-copy and multicopy transformants. Strains coexpressed under pICL1-control the codon-optimized P450 2D6syn (**A**) or P450 3A4syn (**B**) in combination with the wild-type human or *Y. lipolytica* NADPH-P450 reductases (hCPRwt, YlCPR) from integrated single-copies or multiple vector copies as indicated, except the control strain YL23 on the left in A and B, containing only one endogenous chromosomal YlCPR copy, which is present as background in all strains. Cultivation at 28°C in YNBG on glucose, induction by 1% ethanol, microsomes isolation, whole-cell CYP activity and cytochrome P450 reductase (CPR) activity (determined as NADPH-cytochrome c reductase, NCR) measurements were performed as described in Materials and Methods. Filled bars, CPR activity; hatched bars, CYP activities; -, no genes; + genes in single copy; ++ genes in multicopy.

For both CYP2D6 and CYP3A4, the use of the p64D-derived multicopy integration vectors led to a significant increase of expression (Figure 3) and whole-cell CYP activity compared to single copy integrants. CYP2D6 multicopy gene integration gave ~ 70 fold increase of activity towards bufuralol, and multiple copies of the CYP3A4 coding sequence resulted in a ~ 50 fold increased activity towards progesterone (Figure 4). This increase was similar to that observed by overexpressing a CYP1A1 gene in the *Y. lipolytica* strain PO1d [22]. The highest expression levels assessed by CO-spectra for human liver cytochrome P450 2D6 and 3A4 were 92.3 ± 9.2 and 60.9 ± 5.9 pmol per mg microsomal protein, respectively. No results from CO spectra have been reported for CYP1A1 expression in *Y. lipolytica*. Expression levels in the range of 90 and 51–250 pmol per mg microsomal protein have been reported for CYP2D6 and CYP3A4 in bakers yeast, respectively [12,52,53]. 

### Coexpression of CYPs and CPRs

It has been shown that the coexpression of a functional CPR is important for optimal CYP activity [54,55]. In some cases the proteins of the native electron transfer chain can be replaced by other proteins. For example, the adrenodoxin homologue of *Schizosaccharomyces pombe* etp1fd was shown to be able to replace mammalian adrenodoxin and transfer electrons from adrenodoxin reductase to CYP11B1 and CYP11B2 [56]. However, in most cases the mammalian CPRs are more effective in supplying electrons to mammalian CYPs than other CPRs [57]. This suggests that the intrinsic yeast CPRs may be a limiting factor in the heterologous expression and application of mammalian CYPs in yeast. Therefore, we coexpressed not only the endogenous *Y. lipolytica* CPR but also the human CPR.

Cytochrome P450 reductase activity measured as NADPH cytochrome c reductase activity was detected in all clones expressing either human CPR or *Y. lipolytica* CPR, while a control strain transformed with the empty vector showed only low endogenous CPR activity. The multicopy effect described for the hydroxylation activity was also observed for the CPR activity towards cytochrome c (Figure 4).

Multicopy transformants YL11 and YL18 over-expressing the endogenous *Y. lipolytica* CPR showed 7-fold or 40-fold higher activity towards cytochrome c compared to multicopy transformants Yl21 and Yl22 over-expressing the human CPR, respectively. This may be due to a higher affinity of the *Y. lipolytica* CPR to cytochrome c as compared to the human CPR. However, the more likely reason might be the higher YlCPR expression level, as indicated by the more intense band on the Western blot when compared to the human CPR (Figure 3). Despite the higher activity of over-expressed YlCPR towards cytochrome c, YlCPR coexpression did yield much lower CYP activities than achieved with clones coexpressing the human CPR (Figure 4). This indicates that coupling of the human CYPs with *Y. lipolytica* CPR, although possible, is inferior to coupling with human CPR. This is in accordance with earlier results suggesting that mammalian CPR is more effective in transporting electrons to mammalian CYPs as compared to alternative CPRs [3,56,58].

### Determination of operation parameters

Resting and growing whole-cells can be employed as catalysts for biotransformations. Growing cells are considered more favorable than resting-cells when expressing a protein with low stability, since they permit sustained protein expression during biotransformation. However, resting-cells have the advantage that the desired reaction can be investigated independently of growth phenomena and at higher cell densities. Furthermore, biotransformation conditions can be chosen independently from growth conditions minimizing side reactions and allowing identification of potential limitations [59].

Differences between growing and resting cells on bufuralol and protesterone hydroxylation rates were investigated for *Y. lipolytica* harboring hCPR combined with CYP2D6 and CYP3A4, respectively. The hydroxylation rates were determined as product formation rates measuring the concentrations of hydroxybufuralol or hydroxyprogesterone, respectively. Resting-cells showed significantly higher hydroxylation rates than growing cells (data shown in Additional file 1). Therefore, resting-cells were used further in this study.

**Figure 5:**
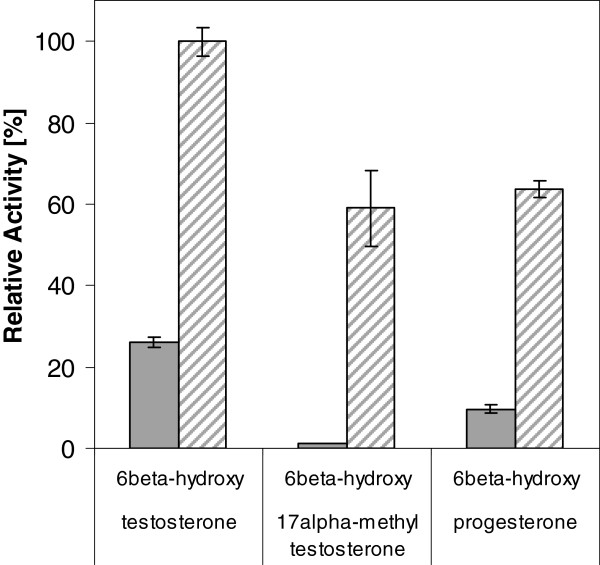
**Whole-cell hydroxylation of different steroids.** Whole-cell hydroxylation of different steroids in aqueous phase system, employing *Y. lipolytica* YL21 and YL22 cells harboring CYP2D6 (filled bars) or CYP3A4 (striped bars), respectively. Data is shown as activity relative to the highest measured apparent hydroxylation rate of testosterone (3.9 ± 0.1 mU per g CDW). Compounds were converted by cells equivalent to ~ 10 g/L CDW for 16 hours. Activity was determined in test tubes as product formation rate after adding steroids dissolved in DMSO to a final concentration of 2 mM.

Both CYP2D6 and CYP3A4 have also been tested for their ability to hydroxylate testosterone, 17-alpha-methyltestosterone and progesterone. CYP2D6 usually prefers substrates containing a basic amine function [3]. Nevertheless, some studies have shown that CYP2D6 is also involved in steroid hydroxylation [60,61]. Both recombinant human liver cytochrome P450 whole-cell *Yarrowia* catalysts hydroxylated testosterone, 17alpha-methyltestosterone and progesterone (Figure 5). Although expression levels have been lower for CYP3A4 compared to CYP2D6, *Y. lipolytica* cells harboring CYP3A4 and hCPR in all cases showed the expected several-fold higher activity towards steroids, with a preference towards testosterone. The untransformed control strain YL23 showed no formation of any hydroxylated steroid. However, *Y. lipolytica* is oxidizing the hydroxy function of testosterone at position 17 to the corresponding keto-function giving androstenedione [62,63]. Such undesired side reactions may hamper the reliability of activity measurements. Therefore, testosterone was excluded and progesterone, another known standard substrate for CYP3A4, was used for bioconversion studies. Furthermore, progesterone was used to investigate the potential of two-liquid biphasic systems and *Y. lipolytica* catalysts for more efficient whole-cell bioconversions. 

### Whole-cell conversion in biphasic systems

Most of the typical human liver CYP substrates are very hydrophobic. Hence, one main function of CYPs is to incorporate a hydroxyl function to render the compound more water soluble and to activate it for further metabolization. Alkane-utilizing yeasts, such as *Y. lipolytica*, can excrete surfactants and adjust their cell surface and cell wall to directly interact with water immiscible organic substrates, like alkanes, long-chain fatty acids and triglycerides, facilitating their uptake [40,41,64-67]. These properties as well as metabolic adaptations and the subcellular organization of the alkane-utilizing yeast cell are obviously supporting the high in vivo turnover numbers observed for the host-own P450s of the CYP52 family involved in primary alkane and fatty acid oxidation [40,41,67-69].

In this study, we investigated whether the unique properties of *Y. lipolytica* will support also the function of heterologously expressed CYPs in this yeast and will give this organism an advantage in CYP-catalyzed bioconversion of hydrophobic substrates dissolved in organic solvents. A spectrum of 10 water immiscible organic solvents ranging from (i) solvents with known good solvent properties (toluene, 1-octanol), (ii) inert fluids (bis-ethyl hexyl phthalate, dibutyl phthalate) to (iii) potential carbon sources (1-decanol, 1-dodecanol, methyl laureate, ethyl oleate, n-decane, n-dodecane) [70,71] were chosen. The solvents toluene and 1-octanol, which are known to be toxic for microorganisms, enabled the highest solubility of steroids. Indeed, they showed the expected toxic effect on *Y. lipolytica* cells, too. Two solvents, known to be utilized as a carbon source by *Y. lipolytica*, n-decane and n-dodecane, conferred very low solubility of progesterone and were therefore excluded from further analyses. 1-decanol, 1-dodecanol, methyl laureate, ethyl oleate, bisethylhexylphthalate (BEHP) and dibutyl phthalate (DBP) had no obvious toxic effects and enabled reasonable solubility of steroids (Additional file 2).

As mentioned before, exposing *Y. lipolytica* cells to water immiscible organic solvents triggers changes in cell structure resulting in increased hydrophobicity [66]. Even when the organic solvent is not utilized by *Y. lipolytica*, morphological changes of the cells immediately become obvious. The cell suspensions become less homogenous and cell agglomerates form. It has been reported that significant changes to the cell surface (increased cell wall hydrophobicity, occurrence of protrusions and special channel like structures) are important for the cells to attach to the hydrophobic substrates as well as for the uptake of these substrates [40,41,66,72,73]. *Y. lipolytica* was observed to adsorb to the organic solvent droplets of DBP and ethyl oleate, similarly to the previously observed adsorption to hexadecane when cells are grown in media containing that alkane as sole carbon source [40,41].

**Figure 6:**
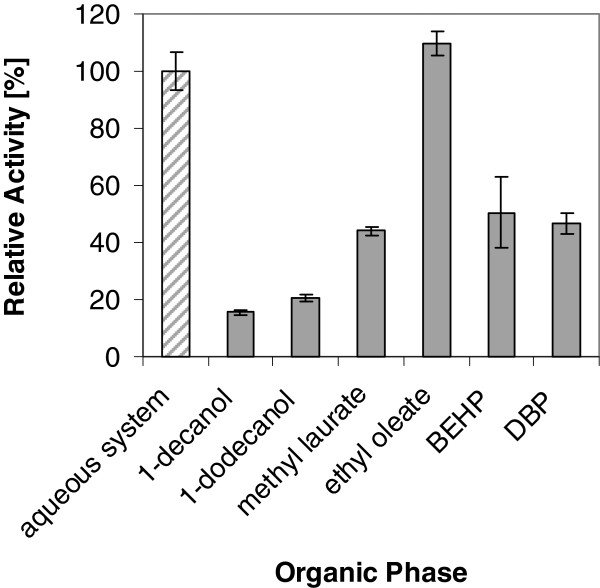
**Whole-cell conversions of *Y. lipolytica*in biphasic conditions.** Comparison of whole-cell conversion with resting *Y. lipolytica* YL22 cells harboring CYP3A4 and hCPR in aqueous (striped bar) and biphasic systems (filled bars) using different organic solvents as 2^nd^ phase. Activity was determined as product formation rate.

Based on the experiences with aqueous whole-cell bioconversions, biotransformations in biphasic systems also were performed with resting-cells. Cells were taken after 26 h of ethanol induction and resuspended in 100 mM potassium phosphate, pH 7.4, containing 1% w/v glucose to a biomass concentration of ~ 12 g/L CDW. The cell suspension was then mixed with an equal volume of the organic phase containing 20 mM of progesterone. Conversions were performed at 30°C over night while shaking. All data were calculated with respect to the aqueous phase volume. Based on other reported experiences [24] for conversion in aqueous systems steroids were added above their solubility limit as DMSO stock solution resulting in precipitation of most of the hydrophobic substrate. Surprisingly, most of the tested water immiscible organic solvents led to a decrease in conversion rate of at least 50% compared to the pure aqueous system where just the organic solvent of the pre-solubilized substrate was added. Only ethyl oleate enabled a slightly increased conversion rate (Figure 6). 

Thus, ethyl oleate was identified as the most promising water immiscible organic solvent for the biphasic conversion of progesterone in shake flask cultures. Comparing one utilizable and one non degradable organic solvent, ethyl oleate and dibutyl phthalate [Mauersberger et al. unpublished results] [74], respectively, were chosen to perform bioconversions under more controlled conditions in bioreactors.

### Two-liquid phase bioreactor process

**Figure 7:**
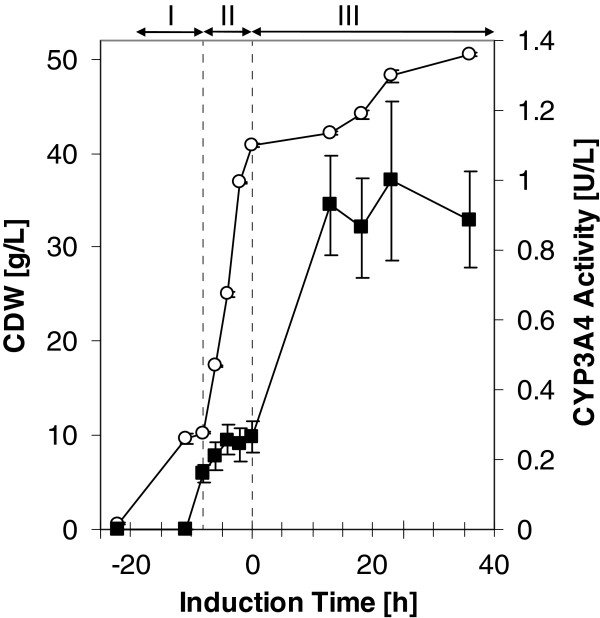
**Biomass and CYP3A4 activity during cultivation of *Y. lipolytica*YL22 in bioreactor.** Time course of biomass concentration and volumetric whole-cell CYP3A4 activity determined during fed-batch cultivation and ethanol induction. Open symbols, biomass concentration determined as cell dry weight; closed symbol, CYP3A4 expression levels measured as volumetric whole-cell activity of 6beta-hydroxyprogesterone formation determined by separate activity assays in test tubes. The Roman numerals show the following process phases: I) batch growth with glucose; II) fed-batch growth over 10 h with exponential addition of glucose at an exponentially increasing feed rate; III) induction phase of about 36 h with a low linear addition of ethanol.

Biphasic and aqueous bioconversions of progesterone with recombinant *Y. lipolytica* catalysts harboring CYP3A4 and hCPR have been performed in a stirred tank bioreactor. For biomass production, a standard batch was run for 16 h (phase I) followed by a fed-batch run for 10 h at a constant growth rate of μ = 0.18/h (phase II). Subsequently CYP3A4 and hCPR production was induced by a low, linearly increasing feed of ethanol over 36 h (phase III). Samples were taken at different time points and specific 6-beta-hydroxyprogesterone formation was determined in test tubes. Interestingly, a low CYP activity was already observed during fed-batch (phase II) on glucose. This reflects a derepression of the pICL1 promoter under conditions of glucose limited growth or glucose depletion. The highest specific 6-beta-hydroxyprogesterone activity of 22 mU/g CDW was reached after roughly 12 h of ethanol-induction and remained constant during the induction time. However, the highest volumetric progesterone hydroxylation rate of 1 U/L (calculated from the specific activity) was reached after roughly 24 h of induction (Figure 7). Cells were harvested after 36 h of induction and resuspended in glucose containing potassium phosphate buffer to a biomass concentration of ~ 25 g/L CDW. Aqueous and biphasic bioconversions in 1.5 L stirred tank reactors used the same cell suspension as starting material. Glucose at a low constant feed of 0.007 g/h/g CDW compared to exponential feed of 1.05 g/h/g CDW during fed batch, was provided as energy source for cell maintenance and regeneration of cofactors.

**Figure 8:**
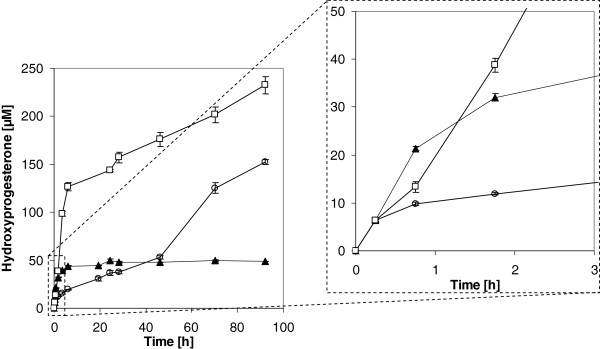
**Hydroxyprogesterone formation during whole-cell conversion in bioreactor.** Time course of hydroxyprogesterone formation during whole-cell conversion of progesterone by *Y. lipolytica* YL22 cells harboring CYP3A4 and hCPR. Closed triangles: aqueous phase system; open circles: with dibutylphthalate (DBP) as second organic phase; open squares: with ethyl oleate as second organic phase.

During the first hour in both biphasic systems less hydroxyprogesterone was formed than in the aqueous system. As of 2 hours of conversion the ethyl oleate based biphasic system provided the highest conversion rates (Figure 8). However, after 6 h the product formation rate of all systems started to drop significantly. While the product formation of the aqueous system came even nearly to a halt after 6 h. The product formation of both biphasic systems continued at a reduced rate throughout the whole biotransformation experiment (Figure 8).

In the beginning of the bioconversion of the different systems the substrate is probably taken up directly from the aqueous phase. The discrepancy in the hydroxylation rate of the different bioconversion systems in this early phase of conversion, is likely due to the difference in availability of progesterone in the aqueous phase. Indeed, for ethyl oleate 8 times and for dibutyl phthalate 42 times higher concentration of progesterone were measured in the aqueous system compared to the aqueous phases of the respective biphasic system. The available substrate concentrations in the aqueous phases stayed more or less constant during the whole bioconversion process excluding the influence of partitioning of substrate on observed variations of bioconversion rates during the conversion process. *Y. lipolytica* cells most probably need some time to adjust to the new hydrophobic environment. This seems to be significantly faster for the naturally occurring utilizable ethyl oleate giving much faster product formation compared to the inert non degradable dibutylphthalate. The generally lower conversion rates in biphasic systems might be explained by lower substrate concentration in the aqueous phase in presence of organic solvents.

Subsequently, the uptake of bioconversion substrate may be achieved in different ways; either *Y. lipolytica* cells continue to take up the low concentrations of substrate from the aqueous phase, or a direct uptake of substrate from the organic phase takes place. Although, the ethyl oleate might be cleaved by secreted lipases, the most reasonable explanation would be the co-uptake of ethyl oleate and dissolved steroid, similarly to the alkane uptake. In such a case, the conversion rate might be limited by the uptake rate of the organic phase.

Although dibuthylphtalate was shown to be a very well suitable substrate for the conversion of hydrophobic substrates in biphasic system using another alkane utilizing yeast [75], in our study dibutyl phthalate as second phase enabled significantly lower conversion rates compared to ethyl oleate.

After approximately 1.5 and 43 h, the overall product formation of both, the ethyl oleate and DBP biphasic systems, respectively, exceed the aqueous system, with an ongoing catalyst activity within the evaluated period of 100 h of bioconversion. However, it was observed that surprisingly the use of DBP as organic phase also lead to a significant change in the product profile formed from progesterone, while ethyl oleate did not. Already after 2 h there is a discrepancy in the hydroxylation pattern in the dibutyl phthalate biphasic system, compared to the aqueous and the ethyl oleate biphasic system. One might speculate that a different extent of emulsification and transport effects in different solvent systems might contribute to this effect. However no experiments were performed to proof this hypothesis. At the beginning, 6-beta-hydroxylation is dominant. However, the hydroxylase specificity is shifted to the formation of an unknown hydroxy product. After roughly 4 hours, the hydroxylase rate towards the unknown position exceeds the rate towards the 6-beta-position. At the end of the two-liquid phase bioreactor experiment the hydroxylation pattern is considerable shifted. The unknown product makes up 65% of the total product compared to only traces in aqueous system. By comparing the unknown product to in-house available hydroxyprogesterone standards 6-beta, 11-alpha, 16-alpha and 17-alpha hydroxyprogesterone, we were not able to identify the new product. Unfortunately the concentration was not high enough to identify the product by other means e.g. NMR.

Oleate esters naturally occur in the membranes of *Y. lipolytica*. They are incorporated into lipids or degraded as energy source and might show less influence on cellular membranes or membrane bound proteins than dibutyl phthalate which is not degraded by *Y. lipolytica*. Furthermore, DBP is more similar to steroids and may enter the binding pocket of CYP3A4 which might explain the distorted hydroxylation patterns.

## Conclusion

The *Y. lipolytica* strain H222-S4 was engineered to express the human liver CYP2D6 and CYP3A4. Co-expressing human cytochrome P450 reductase as well as increasing the copy numbers of both genes improved the catalytic performance of these whole-cell biocatalysts. However the human CYP reductase was the more efficient redox partner. Our results support previous findings, stating that not only human CYP3A4 but to some extent also CYP2D6 is able to hydroxylate steroid molecules such as testosterone, 17α-methyltestosterone and progesterone which are non-typical CYP2D6 substrates.

By using bio-degradable ethyl oleate in a two-liquid biphasic setup instead of aqueous systems, we could show that *Y. lipolytica* cells harboring human CYP3A4 and human CPR can be applied as a whole-cell system with increased biocatalytic stability. This might be caused by different cellular adaptation mechanisms to the organic compounds such as cell wall adaptation or by different transport mechanisms. A systems biology approach might help to clarify these issues, which was out of scope of this study. As expected the alkane-assimilating yeast *Y. lipolytica*, coupled with above described expression strategies, demonstrated to be promising tools for biotransformations of hydrophobic substrates in two-liquid biphasic systems. Especially organic solvents which can be efficiently taken up and/or metabolized by the cell might enable more efficient bioconversion as compared to aqueous systems and even enable simple, more stable, continuous or at least high yield long time processes.

## Materials and Methods

### Chemicals and reagents

Unless stated otherwise, all chemicals were purchased from BD Bioscience (USA), Sigma–Aldrich (Germany), Carl Roth GmbH (Germany), J.T. Baker (Netherlands) and Roche Diagnostics (Germany) at the highest purity available. Oligonucleotides were purchased from IDT (Belgium).

### Strains, media and culture conditions

*E. coli* Top10 or *E. coli* Top10F’ (Invitrogen Corp.) strains were used as host for recombinant plasmid propagation. Cells were grown on Luria-Bertani Agar (Carl Roth GmbH) supplemented with 100 μg/mL ampicillin at 37°C. DNA isolation and purification was done using Fermentas Miniprep kit or Promega Wizard SV Gel isolation and PCR cleanup kit.

In this study, the *Y. lipolytica* strain H222-S4 (*MATA ura3-302*) [76] was used as recipient strain for integration of plasmids described in Table 2. The *Y. lipolytica* strains were grown at 28°C on solid complete medium YPD or on minimal medium YNB (Difco, BD Biosciences, USA) supplemented with 2% w/v glucose (YNBD) and 15 g/L agar [48]. Transformants were selected on solid YNBD (15 g/l agar). Cultivation in liquid media was performed with 200 or 250 mL of buffered minimal YNB medium supplemented with 0.6% w/v glucose and 200 mM potassium phosphate buffer, pH 6.5, in 1 L Erlenmeyer flasks at 28°C. 

**Table 2:** Plasmids used

**Plasmid**	**Description**	**Marker gene (selection in yeast)**	**Reference**
p64ICL1	Parental vector for multicopy integration in *Y. lipolytica*	ura3d4 (mc)^a^	[51]
p65ICL1	Parental vector for single copy integration in *Y. lipolytica*	ura3d1 (sc) ^a^	[51]
p67RYL	Source for *Y. lipolytica* cytochrome P450 reductase (YlCPRwt)	ura3d4 (mc)^a^	[22,49]
pNMTS-CYP-OR-spe	Source for codon optimized human cytochrome P450 3A4 (3A4syn)		Weis et al., unpublished (IMBT, TU Graz)
pBdpTrcRed-3A4(192v)wtA305S	Source for codon optimized human cytochrome P450 reductase (hCPRsyn)		Weis et al., unpublished (IMBT, TU Graz)
ID7262313	Source for cDNA wild-type human cytochrome P450 reductase (hCPRwt)		BioCat, Germany
Cloning vectors:			
pJet1.2	*E. coli* cloning vector		Fermentas, Germany
p64D-linker	Cloning and basic integrative multicopy vector	ura3d4 (mc) ^a^	This work
p65D-linker	Cloning and basic integrative single copy vector, negative control	ura3d1 (sc) ^a^	This work
Integrative vectors for CPR and CYP expression in *Y. lipolytica*:			
p64D-hCPRwt-2D6syn	Multicopy coexpression of human wild-type CPR (hCPRwt), codon optimized CPR (hCPRsyn) or *Y.lipolytica*-own CPR (YlCPR) in combination with codon optimized CYP2D6 or codon optimized CYP3A4, respectively.	ura3d4 (mc) ^a^	This work
p64D-hCPRsyn-2D6syn			
p64D-hCPRwt-3A4syn			
p64D-YlCPR-2D6syn			
p64D-YlCPR-3A4syn			
			
p65D-hCPRwt-2D6syn	Single copy coexpression of human wild-type CPR (hCPRwt) or *Y. lipolytica*-own CPR (YlCPR) in combination with codon optimized CYP2D6 or codon optimized CYP3A4, respectively.	ura3d4 (mc) ^a^	This work
p65D-hCPRwt-3A4syn			
p65D-YlCPR-2D6syn			
p65D-YlCPR-3A4syn			

^a^*URA3* marker genes for single copy (sc, *ura3d1*) or multicopy (mc, *ura3d4*) selection with different promoter lengths.

All seed and inoculum cultures were prepared using buffered minimal medium YNB (Difco) supplemented with 1% w/v glucose and 200 mM potassium phosphate, pH 6.5. The defined mineral medium used for the main fed-batch cultures contained per liter.

6.0 g (NH_4_)_2_SO_4_, 2.0 g KH_2_PO_4_, 0.24 g K_2_HPO_4_, 1.4 g MgSO_4_ x 7 H_2_O, 0.6 g NaCl, 0.58 g NaNO_3_, 0.38 g CaCl_2_, 1 mL PTM trace element solution, i.e. 0.5 g/L H_3_BO_3_, 0.04 g/L CuSO_4_ x 5 H_2_O, 0.1 g/L KI, 0.303 g/L MnSO_4_ x 1 H_2_O, 0.2 g/L Na_2_MoO_4_ x 2 H_2_O, 0.4 g/L ZnSO_4_ x 7 H_2_O, 0.2 mL of ethanolic iron chloride solution i.e. 30 g/L FeCl_3_ x 6 H_2_O, 1 mL thiamin hydrochloride solution i.e. 4 g/L and 16.5 g glucose monohydrate.

The feed solution contained 550 g glucose monohydrate per liter, 3 mL PTM trace element solution, 0.2 mL ethanolic iron chloride solution and 3 mL thiamin hydrochloride solution.

For induction of the pICL1-controlled CYP expression, pure ethanol was used as feed solution.

### Genes and vectors

The integrative multicopy and single copy *Y. lipolytica* vectors p64ICL1, p65ICL1 [51,77,78] were adapted by replacing the *ICL1* intron and gene with a small linker containing *Spe*I and *Asc*I restriction sites and used for cloning and co-expressing CYPs and CPRs. The vector p67RYL was used to isolate the *Y. lipolytica* CPR gene which is essentially the wild-type gene but alanine at position 2 was chanced to proline [48,79,80]. pNMTS-CYP-OR-spe and pBdpTrcRed-3A4(192v)wtA305S were used to isolate codon optimized human CYP3A4 and codon optimized human CPR gene, respectively. Both were optimized for expression in yeast, i.e. hybrid optimized for *P. pastorisS. pombe* and *S. cerevisiae* (Weis et al., unpublished).

Wild-type human CYP2D6 (ID30915411), wild-type human CYP3A4 (ID7262313) and wild-type human CPR (ID3882411) genes were isolated from cDNA clones (BioCat GmbH, Germany).

For the codon optimization of the CYP2D6 gene, the free software “Gene Designer V1.1.4.1” (DNA 2.0, USA) was used to design a gene optimized for expression in yeast, i.e. hybrid optimized for *P. pastoris* and *Y. lipolytica*, and then synthesized.

### Vectors constructions

For vector construction, standard molecular biology procedures were performed [81]. The multicopy vector p64D-linker and single copy vector p65D-linker were obtained by replacing the isocitric lyase 1 (*ICL1*) gene and intron with a linker containing *Spe*I and *Asc*I site, in the parental vectors p64ICL1 and p65ICL1, respectively. Both vectors contain the URA3 selection marker. The p64-vector variants contain a deficient, truncated *ura3d4* promoter as multicopy selection marker, which gives only sufficient amounts of gene product when several copies (at least 8–10) of the vector are integrated into the genome. This allows for selection of clones with multicopy integrations or gene multiplications. The p65-vector variants contain the *ura3d1* promoter sufficient for single copy selection [47,48].

Co-expression of single and multicopy vectors with combinations of different CPRs and cytochrome P450s were obtained by cloning overlap extension PCR products into the *Spe*I and *Asc*I sites (Figure 1). Each gene was placed under the control of the *ICL1* promoter [82]. Shortly; each fragment, i.e. CPR, *ICL1* promoter and terminator, CYP was amplified via PCR using primers with overhangs homologous to adjoining parts. Overlap extension PCR was done in two steps. All fragments were added to the first reaction mix PCR was run for 20 cycles using Phusion Polymerase (NEB). Then flanking primers were added to the reaction which was run for another 25 cycles to amplify the overlap construct. The fuel-length PCR construct was then cloned into pJet1.2 using the CloneJET™ PCR Cloning Kit (Fermentas) and verified by sequencing (LGC genomics, Germany). Confirmed inserts were cloned with SpeI/AscI into p64D-linker or p65D-linker, respectively, using T4 DNA ligase (Fermentas) according to standard procedure.

### Transformation into Y. lipolytica

Transformation of *Y. lipolytica* H222-S4 was performed by the lithium acetate method [38] or by electroporation according to a condensed protocol [83]. All vectors contained the rDNA (ribosomal DNA fragment) of *Y. lipolytica* as integration site and were digested by *Sac*II before transformation. The transformants were selected for Ura^+^ phenotype on minimal YNBD medium (2% w/v glucose). Colonies appearing after 2–3 days (single copy) and 2–3 weeks (multicopy) were transferred onto fresh plates and sub-cultured. The resulting prototrophic recombinant *Y. lipolytica* strains used for CYP and CPR expression and steroid biotransformation studies and their estimated integrated vector copies are given in Table 1.

Real-time PCR (RT-PCR) was used to estimate the copy number of the integrated expression cassettes. Genomic DNA was isolated from *Y. lipolytica* transformants grown overnight in minimal YNB medium supplemented with 1% w/v glucose (YNBD) [84]. The *ICL1* set of primers, ICL1-fw (5′-CCA GCA GCC CGA GAT TGA-3′) and ICL1-rv (5′-ACT CAG CAC CGG ACC ACT TC-3′), anneal to the single copy of the endogenous *Y. lipolytica ICL1* gene within the chromosome. The Amp primers, Amp-fw (5′-GCT ATG TGG CGC GGT ATT ATC-3′) and Amp-rv (5′-GTA TGC GGC GAC CGA GTT-3′), target the *amp*^*R*^ marker gene present on the integrated vectors. *Y. lipolytica* YL23 was used as a control organism with a single copy of both the *ICL1* and *amp*^*R*^ target sequences. Reaction mixes of 18 μl consisted of 100 pg template DNA, Power SYBR Green Master Mix (Applied Biosystems, CA, USA), and 250 nM of each primer. Each reaction was run in duplicate in an ABI PRISM 7300 Real Time PCR machine (Applied Biosystems, CA, USA). The profile used was, 95°C for 10 min, fallowed by 40 cycles of (95°C for 15 s, 60°C for 60 s). Data collection was done after each 60°C step. A melting curve analysis was conducted after the amplification, heating from 45°C to 95°C. Analysis was done using Sequence Detection Software SDS (Applied Biosystems, version 1.2). Average Ct values of the 2 profiles (*ICL1* and Amp) were used to estimate the relative copy number for the selected transformant [85].

### CPR and CYP induction

*Y. lipolytica* clones were grown in YNBG (1% w/v glucose) overnight at 28°C and 220 rpm. Exponentially growing cells were taken to inoculate the YNBG (0.6% w/v glucose) main culture to a starting OD_600_ of ~ 0.5. The main culture was grown at 28°C and 220 rpm for 17 – 20 h until glucose was fully consumed. After additional 2–4 h, the expression of CPR and CYP under the control of the *ICL1* promoter was induced by adding ethanol to a final concentration of 1% v/v. Additional 1% v/v ethanol was added after ~ 8 h and ~ 20 h. After 24 h the cells were harvested by centrifugation and further used for either whole-cell biotransformation assays or microsome isolation.

### Isolation of microsomes

Yeast cells were harvested by centrifugation at 2 000 g for 10 min and washed twice with water. 4–6 g of cells were resuspended in ~ 20 ml disruption buffer (50 mM potassium phosphate, pH 7.9, containing protease inhibitor 1 mM PMSF, 5% w/w glycerol, 1 mM EDTA and 2 mM DTT). A crude cell lysate was obtained by mechanical cell disruption using “Merkenschlager homogenization” [86]. After cell disruption cell debris was removed by centrifugation at 10 000 g and 4°C for 10 min. To pellet the microsomal fraction, the supernatant was centrifuged at 100 000 g and 4°C for 1 h. The membrane pellet was resuspended in disruption buffer to ~ 1 mg pellet per mL using a Dounce homogenizer and pestle.

### SDS-PAGE and Western blot analyses

Total protein content of the microsomal preparation was determined by Bradford using the biorad protein assay kit (Bio-Rad, Germany) before separating the proteins by using the NuPAGE® electrophoresis system (Invitrogen Ltd). Samples containing ~ 20 μg total protein in 15–25 μL loading buffer without reducing agent were incubated at RT for at least 10 min. For separation, a NuPAGE Novex 4-12% Bis-Tris-Gel and MOPS buffer were used. The PageRuler Prestained Protein Ladder (Fermentas) was used as molecular mass calibration standard.

Western blotting was done according to the protocol provided with the MAB-2D6 and WB-3A4 kits (BD Gentest™). On completion of PAGE, the proteins were transferred electrophoretically onto nitrocellulose membrane (GE Healthcare Europe GmbH) in a wet blotting system. Then the membranes were blocked at room temperature overnight with whey powder. The blot was developed by incubation with NBT/BCIP at RT for 5 min.

### NADPH cytochrome c reductase (NCR) activity

The cytochrome P450 reductase-catalysed reduction of bovine heart cytochrome c was measured at 550 nm essentially as described [70]. A 300 μM cytochrome c solution in 50 mM Tris–HCl buffer, pH 7.5, was mixed with 2 to 80 μg of microsomal protein and made up with Tris–HCl to 650 μL. Fifty μL of 50 mM KCN solution, pH 7.7, were added to mask cytochrome c oxidase activity. Reaction was started by adding 50 μL of 1.5 mM NADPH.

Activities were measured on a UV/Vis DU 800 spectrophotometer (Beckman Coulter, USA) and calculated by using ϵ550nm = 21 mM^-1^ cm^-1^ as molar extinction coefficient of cytochrome c.

### Quantification of cytochrome P450

CYP concentrations in the isolated microsomes were determined by reduced carbon monoxide spectra [87]. Six to 12 mg of microsomal protein were added to 100 mM sodium phospate buffer, pH 7.4, containing 20% w/w glycerol to an end volume of 2 mL. One hundred μL 200 mM KCN, pH 7.7, were added to mask the spectral interference of cytochrome oxidase (negative absorption at 445 nm) with the CYP peak at 450 nm in the CO difference spectrum [49]. A few grains sodium dithionite were added to reduce the CYP. The mixture was transferred into polystyrene cuvettes (Sarstedt, Germany) and a reference spectrum was recorded from 400 to 500 nm (Specord 205 UV/Visible spectrophotometer, Analytik Jena, Germany). The mixture was then bubbled with carbon monoxide for 60 s, and the spectrum was measured repeatedly several times.

The CYP concentration was calculated using a molar extinction coefficient of ϵ_450nm_ = 91 mM^-1^ cm^-1^.

### CYP activity of microsomes and whole-cells

*In vitro* substrate conversion with microsomes was performed essentially as described in [88]. Twenty five μM bufuralol or 2 mM steroid (in DMSO) e.g. progesterone, 17alpha-methyltestosterone or testosterone, and 1 mM NADPH were added to 100 mM possium phosphate buffer, pH 7.4, and pre-incubated for 2–3 min at 37°C. The reaction was started by adding 20 μL microsomal preparation (0.6 to 0.8 mg total protein) to give a total volume of 200 μL. The reaction mixture was incubated at 37°C for 20 min (bufuralol) or 1 h (steroid) while shaking and stopped by adding 20 μL 70% perchloric acid. After 20 min of incubation on ice, prednisolone (in DMSO) was added to a final concentration of 50 μM as internal standard and the mixture centrifuged for 10 min at 16 100 g. One hundred μL supernatant were transferred to a fresh microtiter plate and stored at −20°C.

For whole-cell conversions, yeast cultures were spun at 2000 g for 15 min and the cell pellets were resuspended in 100 mM potassium phosphate buffer, pH 7.4, containing 1% w/v glucose to a biomass concentration of ~ 10 g/L CDW. Stock solutions of substrate were added to 200 (eppendorf tube) or 1000 μL (PYREX tubes) of cell suspension. Bufuralol was added as 1 mM stock solution to cell suspension yielding a final concentration of 25 μM. Steroid e.g. progesterone, 17alpha-methyltestosterone or testosterone was added as 100 mM stock solution in DMSO to cell suspension yielding a final concentration of 2 mM. Whole-cell conversion of bufuralol and progesterone was performed at 30°C for 20 – 60 min or several hours while shaking, respectively. Then prednisolone (in DMSO) was added to a final concentration of 50 μM as internal and the reaction was stopped by centrifugation at 16 100 g for 10 min. Supernatants were transferred to fresh reaction tubes and stored at −20°C.

### CYP activity in whole-cell biphasic systems

Twenty to fifty mL yeast cultures were centrifuged at 2000 g for 15 min and the pellet was resuspended either in induction media supplemented with 1% v/v ethanol or in 100 mM potassium phosphate buffer, pH 7.4, containing 1% w/v glucose to a biomass concentration of ~ 10 g/L CDW. An equal volume of organic solvent containing 20 mM progesterone, was added to 1000 μL of cell suspension. The biphasic whole-cell conversions were performed in 10 mL PYREX tubes at 30°C while shaking at 320 rpm for several hours. Reactions were stopped at desired time points and the two phases were separated by centrifugation at 16 100 g for 10 min. Two to five hundred μL aqueous and organic phase were transferred to fresh reaction tube or GC-vial, respectively. Prednisolone (in DMSO) was added to a final concentration of 50 μM as internal standard. An equal volume of isopropanol was added to the organic phase, and samples stored at −20°C.

### Fed-batch cultivation in bioreactors

Cultivation process was performed in a 5 L stirred tank bioreactor (Biostat C, Sartorius, Germany). The initial batch working volume was 3.5 L (minimal mineral salt medium M with initial 1.5% w/v glucose) and was increased to 4 L at the time of harvesting. The set-points of all control variables were maintained during the entire process, thus, the cultivations were accomplished under the conditions of constant temperature of 28°C, 10 L/min air flow (i.e. without any oxygen enrichment), 1500 rpm agitation, pH 5.5. Automated control of the pH was achieved by using 25% ammonia and 25% phosphoric acid solutions. The process consisted of the biomass growth phase on glucose, i.e. batch and exponential fed-batch cultures, and the expression phase on ethanol with linear feed addition. The cultivation started at time 0 h with a batch (1.5% w/v glucose) during which the pO_2_ value continuously decreased and the base consumption increased, i.e. no control of the pO_2_ set-point was applied. After 14–16 h, the pO_2_ increased rapidly and base consumption stopped due to glucose depletion. At this point the exponential addition of glucose feed solution was started and continued over 10 h according to the function f(t) = 1.05*e^0.18*t^ in grams of glucose per hour. During the subsequent production phase, 0.5% v/v ethanol was maintained for 34–36 h in accordance with the function f(t) = 1.05*e^0.002*t^ in grams of ethanol per hour. Biomass concentration was determined by measuring cell dry weight (CDW) e.g. 2 mL cell suspension was centrifuged at 16 000 g for 10 min. Supernatant was discarded and cell pellet dried at 100°C till constant weight. Cells where harvested by centrifugation at RT and 2 000 g for 15 min and cell pellets were resuspended in 100 mM potassium phosphate buffer, pH 7.4, supplemented with 0.5% w/v glucose as energy source to a desired biomass concentration of ~ 25 g/L CDW.

### Resting-cell biotransformation in bioreactor

Biotransformations were performed in a 1.5 L stirred tank bioreactor (DASGIP Parallel Bioreactor Systems, DASGIP BioTools, Germany). The batch working volume was 600 mL (phosphate buffer with glucose and ethanol-induced *Y. lipolytica* cells as indicated above) for aqueous phase system and 450 mL aqueous phase plus 150 mL organic phase for biphasic systems. The set-points of all control variables were maintained during the entire biotransformation at 30°C, 1 NL/min airflow (i.e. without any oxygen enrichment), 500 rpm agitation and pH 7.4.

The transformations began at 0 h by adding 100 mM progesterone stock solution (in DMSO) to a final concentration of 2 mM or 150 mL water immiscible organic solvent containing 20 mM progesterone. Glucose was provided at a low constant rate of 0.17 g h^-1^ over the entire biotransformation time of 90 hour. Samples were taken at different time points and the reaction was stopped removing the cells by centrifugation at 16 100 g for 10 min. Aqueous and organic phase samples were withdrawn as described above.

### Analysis by HPLC-MS

Bufuralol and metabolites were separated by HPLC (1200 series, Agilent technologies, USA) with a XDB-C18, 1.8 μm, 4.6 x 50 mm column (Agilent technologies, USA) using a gradient based on 10 mM ammonium acetate, pH 5.5, and acetonitrile at a flow rate of 0.9 mL/min. Metabolites were detected using MSD SL detector equipped with an electron spray ionization (ESI) unit (Agilent technologies, USA).

Hydroxylated products were quantified by external calibration using reference metabolites or metabolite derivates.

Steroid metabolites were separated by HPLC (1200 series, Agilent technologies, USA) with a Chromolith RP-C18e, 5 μm, 4.6 x 100 mm column (MERCK KGaA, Germany) using a gradient based on water and acetonitrile, both acidified with 0.1% v/v acetic acid, at a flow rate of 1 mL/min.

## Abbreviations

Yl: *Yarrowia lipolytica*; h: human; CPR: cytochrome P450 reductase; CYP: cytochrome P450; DBP: dibuthylphtalat; EO: ethyl oleate; CDW: cell dry weight; NCR: NADPH cytochrome c reductase; *ICL1*: isocitric lyase 1; sc: single copy; mc: multicopy.

## Competing interests

The authors declare that they have no competing interests.

## Author’s contributions

AB, SM, and AG drafted the outline of the expression experiments. BB, AS and AB drafted the outline of the two-phase system and fermentation experiments. SM helped with the selection of expression vectors, strains and cultivation conditions. AB and GM carried out the experiments and analyzed the data. AB wrote the paper which was later revised and corrected by AG and BB. All authors read and approved the final manuscript.

## Supplementary Material

Additional file 1 **Influence of growth phase on whole-cell conversion rates. Comparing Diagrams of whole-cell conversions in aqueous systems by growing and resting cells of *Y. lipolytica*harboring CYP2D6 or CYP3A4, respectively.** Click here for file

Additional file 2 Solubility of progesterone in organic solvents. Solubility of progesterone in the different organic solvents shown as a diagram. Click here for file
