# Supplementary material for: Steroid biotransformations in biphasic systems with Yarrowia lipolytica expressing human liver cytochrome P450 genes
Source: Microb Cell Fact. 2012 Aug 9;11:106. doi: 10.1186/1475-2859-11-106 (PMC3544689; doi:10.1186/1475-2859-11-106)
Supplement: Additional file 1 — Influence of growth phase on whole-cell conversion rates. Comparing Diagrams of whole-cell conversions in aqueous systems by growing and resting cells of Y. lipolyticaharboring CYP2D6 or CYP3A4, respectively. [file 1475-2859-11-106-S1.pdf]

Influence of growth phase on whole-cell conversion rates.

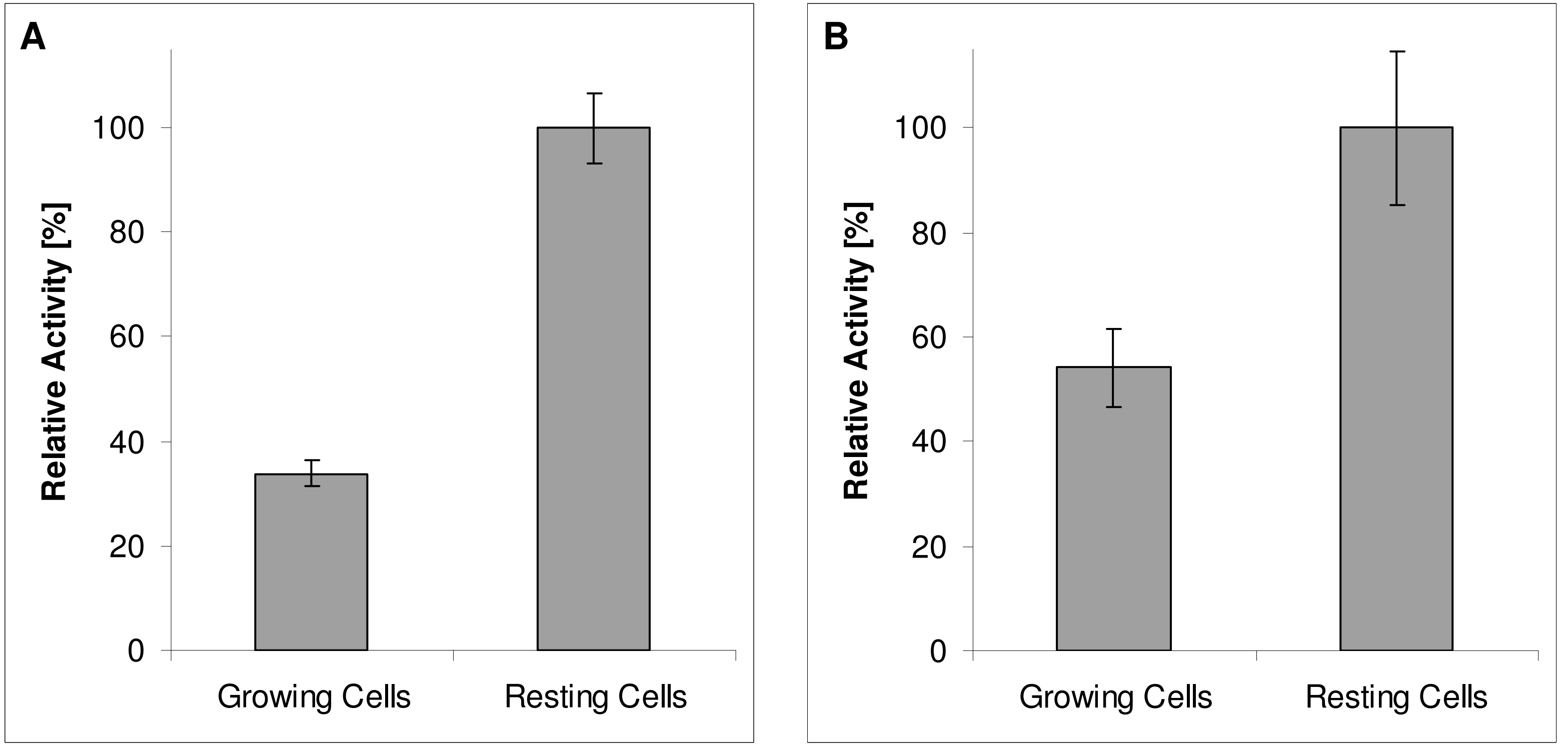

Whole-cell conversions in aqueous systems by growing and resting cells of Y. lipolytica YL21 harboring CYP2D6 and hCPR (A) and YL22 harboring CYP3A4 and hCPR (B), after 26 h of ethanol induction was quantified in triplicate. Incubation time 15-60 min for bufuralol (A) or 30-120 min for progesterone (B) and cell concentrations of 10g/l were used. Activity was determined as product formation rate measuring either hydroxybufuralol or hydroxyprogesterone concentrations.
