# Supplementary material for: Steroid biotransformations in biphasic systems with Yarrowia lipolytica expressing human liver cytochrome P450 genes
Source: Microb Cell Fact. 2012 Aug 9;11:106. doi: 10.1186/1475-2859-11-106 (PMC3544689; doi:10.1186/1475-2859-11-106)
Supplement: Additional file 2 — Solubility of progesterone in organic solvents. Solubility of progesterone in the different organic solvents shown as a diagram. [file 1475-2859-11-106-S2.pdf]

Solubility of progesterone in organic solvents

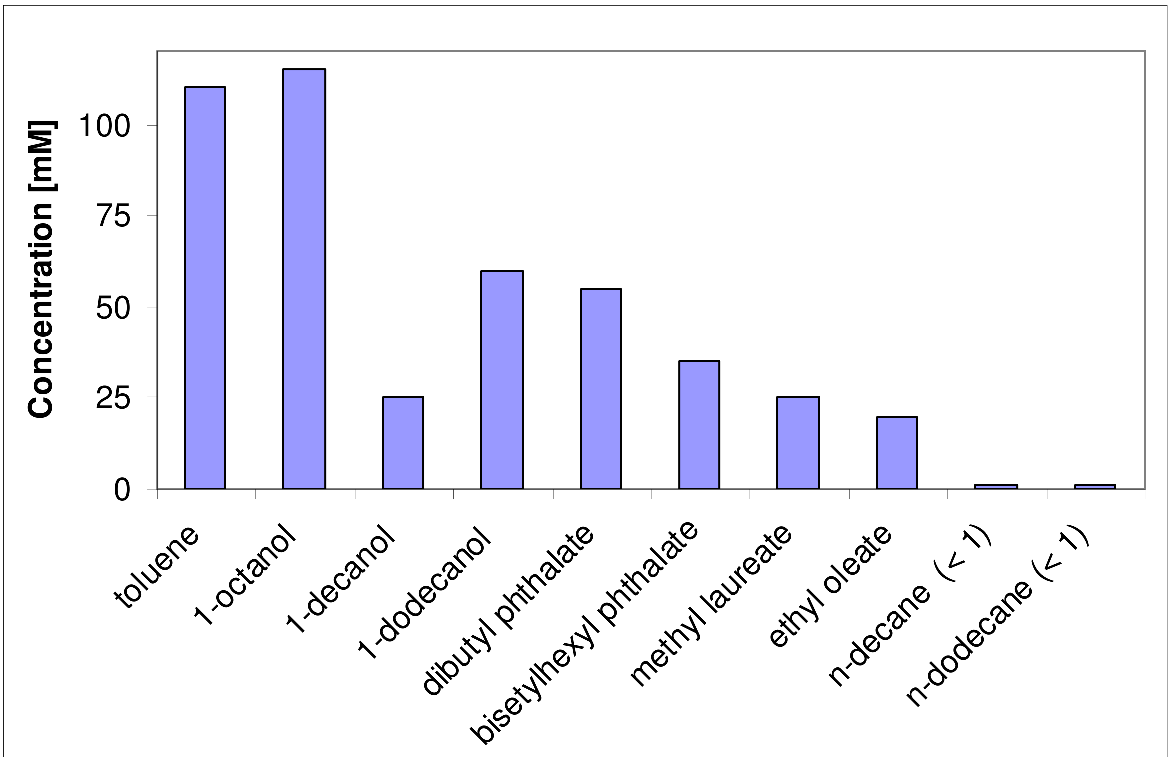

Solubility of progesterone in different organic solvents determined by gradually adding solvents to a certain amount of progesterone till complete dissolution was obtained.
